# The Potential Influence of Advanced Glycation End Products and (s)RAGE in Rheumatic Diseases

**DOI:** 10.3390/ijms24032894

**Published:** 2023-02-02

**Authors:** Charlotte Delrue, Reinhart Speeckaert, Joris R. Delanghe, Marijn M. Speeckaert

**Affiliations:** 1 Department of Nephrology, Ghent University Hospital, 9000 Ghent, Belgium; 2 Department of Dermatology, Ghent University Hospital, 9000 Ghent, Belgium; 3 Department of Diagnostic Sciences, Ghent University, 9000 Ghent, Belgium; 4 Research Foundation-Flanders (FWO), 1000 Brussels, Belgium

**Keywords:** autoimmune inflammatory joint disease, cardiovascular risk, carboxyethyllysine, carboxymethyllysine, methylglyoxal, pentosidine, polymorphisms, (soluble) receptor of advanced glycation end products

## Abstract

Advanced glycation end products (AGEs) are a class of compounds formed by nonenzymatic interactions between reducing sugars and proteins, lipids, or nucleic acids. AGEs can alter the protein structure and activate one of their receptors, specifically the receptor for advanced glycation end products (RAGE). These phenomena impair the functions of cells, extracellular matrix, and tissues. RAGE is expressed by a variety of cells and has been linked to chronic inflammatory autoimmune disorders such as rheumatoid arthritis, systemic lupus erythematosus, and Sjögren’s syndrome. The soluble (s)RAGE cleavage product is a positively charged 48-kDa cleavage product that retains the ligand binding site but loses the transmembrane and signaling domains. By acting as a decoy, this soluble receptor inhibits the pro-inflammatory processes mediated by RAGE and its ligands. In the present review, we will give an overview of the role of AGEs, sRAGE, and RAGE polymorphisms in several rheumatic diseases. AGE overproduction may play a role in the pathogenesis and is linked to accelerated atherosclerosis. Low serum sRAGE concentrations are linked to an increased cardiovascular risk profile and a poor prognosis. Some RAGE polymorphisms may be associated with increased disease susceptibility. Finally, sRAGE levels can be used to track disease progression.

## 1. Introduction

The Maillard reaction, which results from the non-enzymatic interaction of reducing sugars and associated metabolites with proteins, amino acids, and peptides, produces a diverse set of compounds known as advanced glycation end products (AGEs) [1]. A nucleophilic addition reaction between a free amino group from a protein and a carbonyl group from a reducing sugar results in the formation of an unstable, freely reversible Schiff base. When a transition metal is present, this base is rearranged to a more stable intermediate, an Amadori product, which is then oxidized to produce the final fluorescent cross-linking (e.g., pentosidine), non-fluorescent cross-linking (e.g., methylglyoxal lysine dimer (MOLD)) or non-cross-linking (e.g., N^ε^-carboxymethyl-lysine (CML)) AGEs [2,3]. AGEs can also be produced by glucose autoxidation and oxidative stress during chronic inflammation. Previously, a detailed overview of the complex and heterogeneous pathways of chemical formation of AGEs, including several precursors and processes, was published [4]. AGEs alter the three-dimensional integrity of various plasma proteins, which can cause functional abnormalities and contribute to the pathogenesis of a variety of diseases [5].

AGEs bind to the receptor of advanced glycation end products (RAGE), a member of the immunoglobulin superfamily, activating pro-inflammatory responses and promoting inflammatory cell activity [6,7,8]. RAGE, first isolated from the human lung [9], exists in vivo as transmembrane molecules and soluble molecules. The receptor is divided into extracellular, transmembrane, and intracellular segments [10]. RAGE can bind to a variety of ligands due to its unique structure and existing form, including AGEs, S100, calcium/grain, high-mobility group protein 1 (HMGB1), and amyloid-protein (A) [11]. This multiligand receptor, expressed by innate immune cells such as macrophages and granulocytes, as well as endothelial cells, vascular smooth muscle cells, and adipocytes, is involved in pro-inflammatory processes, though its exact function is unknown [12]. In non-inflamed tissues, cell surface RAGE expression is relatively low under physiological conditions, whereas during inflammation, it is up-regulated in response to ligand exposure [13]. The interaction of AGEs with RAGE activates intracellular cells and secretes cytokines such as tumor necrosis factor-alpha (TNF-α), which is important in the inflammatory response [7,14]. AGEs may also bind to oligosaccharyltransferase-48 (AGE-R1), 80K-H phosphoprotein (AGE-R2), galectin-3 (AGE-R3), lectin-like oxidized low-density lipoprotein receptor-1 (LOX-1), macrophage scavenger receptors, fasciclin, epidermal growth factor (EGF)-like, laminin-type EGF-like, and link domain-containing scavenger receptor-1/2 (FEEL1/2) and CD36. It is unknown whether a specific form of AGE preferentially binds to a specific type of receptor or whether ligand and receptor interact tissue-specifically. AGE detoxification and degradation are mediated by AGE-R1, AGR-R3, and macrophage scavenger receptor types I and II [15].

The higher the expected density of the cell-bound receptor, RAGE, the greater the likelihood of increased production of soluble (s)RAGE by cleavage [16]. sRAGE is a positively charged 48-kDa cleavage product that keeps the ligand binding site but loses the transmembrane and signaling domains [17,18]. This soluble receptor inhibits the pro-inflammatory processes mediated by RAGE and its ligands by acting as a decoy [19,20,21]; although, a linkage with pro-inflammatory conditions has also been reported [22]. Because binding of RAGE with its ligand increases RAGE shedding, blood sRAGE concentration may reflect tissue RAGE expression [23]. Aside from the membrane-bound RAGE isoform, two circulating sRAGE isoforms lacking transmembrane and cytoplasmic domains have been identified. The majority of sRAGE (cleaved RAGE or cRAGE) is produced by matrix metalloproteinases (MMPs) resulting in proteolytic cleavage of membrane-bound RAGE, whereas endogenous secretory (es)RAGE (also known as RAGEv1) is produced by alternative splicing of RAGE mRNA [23].

The *RAGE* gene is located on chromosome 6 in the class III region of the major histocompatibility complex locus, and several functional RAGE polymorphisms have been described. The Ser82 allele of the Gly82Ser polymorphism, as well as the A allele of the 2184 A/G polymorphism, appear to have a proinflammatory effect and are linked to the progression of renal disease and cardiovascular disease in diabetes [24,25]. Other polymorphisms, such as the A allele of the 374 T/A polymorphism, are linked to a lower risk of cardiovascular disease [25], whereas Gly allele of Gly82Ser, G allele of 2184 A/G, and C allele of the 429 T/C polymorphism are associated with increased sRAGE concentrations and RAGE expression [26,27,28,29,30].

An overview of current knowledge of the importance of the AGE-RAGE axis in various rheumatic diseases, as well as the influence on the development of cardiovascular disease in these patients, is currently lacking. For these reasons, we will discuss in the present review the potential involvement of AGEs and (s)RAGE in rheumatoid arthritis, systemic lupus erythematosus, Sjögren’s syndrome, adult-onset Still’s disease and juvenile idiopathic arthritis. We searched the PubMed database for articles using the keywords “advanced glycation end products”, “(soluble) receptor for advanced glycation end products”, “rheumatoid arthritis”, “systemic lupus erythematosus”, “Sjögren’s syndrome”, “adult-onset Still’s disease”, and “juvenile idiopathic arthritis”.

## 2. Rheumatoid Arthritis

Rheumatoid arthritis (RA) is a chronic, progressive autoimmune inflammatory joint disease with synovial joint inflammation, synoviocyte overgrowth, cartilage destruction, and systemic extra-articular manifestations [31]. Although the exact cause of RA is still unknown, there is ample proof that it is a polygenic illness with multiple genetic and environmental causes that result in chronic inappropriate inflammatory processes [32,33]. Chronic inflammation can cause oxidative stress and, as a result, the formation of reactive carbonyl compounds, which are partially converted into AGEs [34,35]. In this way, a positive feedback loop of AGE-RAGE interaction is established, maintaining an inflammatory state in which AGEs can be formed [36,37].

### 2.1. The Importance of Advanced Glycation-End Products in Rheumatoid Arthritis

Numerous studies examined AGEs’ role in the pathophysiology of RA patients (Table 1) [38,39,40,41,42,43,44,45,46,47,48,49,50,51,52,53]. A cross-sectional study investigated the association between serum CML and pentosidine and the presence and severity of RA. Serum CML and pentosidine concentrations were significantly higher in RA patients compared to control subjects (*p* < 0.001). Significantly higher serum CML and pentosidine concentrations were found in patients with high disease activity compared to patients with moderate disease activity (*p* < 0.001, *p* = 0.019, respectively). A multiple logistic regression analysis revealed that CML was associated with the presence of RA (OR = 1.21, 95% CI: 1.05–1.39, *p* = 0.006). CML was also independently correlated with disease activity score for 28 joints (DAS28 score) in a multivariate stepwise regression analysis (*p* = 0.001) [40]. A CML cut-off value of 45.2 g/L predicted the presence and severity of RA with 88% sensitivity and 82% specificity. The authors hypothesized that the synergistic effect of increased serum pentosidine and CML could worsen the severity of RA by increasing local glycoxidation and the formation of related cross-links, as well as amplifying an inflammatory phenomenon [40]. Pentosidine also showed a strong, positive connection with erythrocyte sedimentation rate (ESR) (r = 0.226, *p* = 0.011) and C-reactive protein (CRP) (r = 0.785, *p* < 0.0001) in a study of 133 nondiabetic patients with RA [39]. In a small cross-sectional trial, rabbit-anti-CML-IgG and goat-anti-rabbit-IgG were used to treat frozen synovial tissue samples. CML was seen in the synovial lining, sublining, and endothelium. Some macrophages (CD68^+^) and T-cells (CD45RO^+^) in RA also immunostained positively for CML, but B-cells did not. A role for CML was suggested in the pathogenesis of RA by the different immunostaining pattern between RA and osteoarthritis, as well as the presence of CML on macrophages and T-cells. This might be a result of the introduction of novel epitopes that can support or even start an autoimmune response [41].

Another cross-sectional trial showed significantly higher serum methylglyoxal (MGO) concentrations in RA patients versus control subjects (*p* < 0.001), as well as higher levels in RA patients with higher disease activity versus those with moderate disease activity (*p* = 0.019). MGO was independently associated with the presence of activity disease in RA, according to multiple logistic regression analyses (OR = 1.17, 95% CI: 1.02–1.31, *p* = 0.01) [42]. MGO may induce and aggravate the severity of RA through a process involving nuclear factor-kappa B (NF-κB) activation [43,44]. Higher average RAGE and transthyretin plasma concentrations, as well as glycation, were measured in RA patients and showed a significant positive correlation. Tranthyretin–RAGE interaction may imply the potential to exert inflammatory signaling cascade activation via RAGE in RA-fibroblast-like synoviocytes and increase gene expression, particularly in RA peripheral blood mononuclear cells, indicating a key role in inflammation. TNF-α stimulation is also important in promoting transthyretin and RAGE expression dysregulation [45]. Studies utilizing anti-TNF medication as an antioxidant provide evidence for the role of TNF in the pathophysiology of RA [54]. Twenty-two RA patients received etanercept therapy for a total of six months. At the end of the treatment period, serum total pentosidine levels decreased significantly (*p* < 0.05). In RA patients, urinary total pentosidine levels were positively correlated to the DAS28 (r = 0.395, *p* < 0.05), and serum total pentosidine levels correlated with the number of swollen and painful joints (r = 0.449, *p* < 0.05). According to this study, etanercept seems to inhibit the development of pentosidine, oxidative DNA damage, and lipid peroxidation in RA patients [54].

Autofluorescence (AF) levels in the skin of 93 patients with RA and 43 healthy volunteers were evaluated to determine the effectiveness of assessing skin AGE in individuals with bone and joint illnesses. Compared to healthy controls, patients with RA showed significantly higher AGE AF levels in the skin (median: 2.16 and range: 1.45–2.94). Patients with severe joint destruction also tended to have higher AF (median: 2.127 and range: 1.45–2.94) than those with little joint destruction (median: 2.127 and range: 1.55–2.68) or moderate destruction (median: 2.120 and range: 1.50–2.82), although the difference was not statistically significant [46].

In RA, autoantibodies that target age-damaged IgGs are linked to the autoimmune response in RA [55,56,57,58,59,60,61]. In 105 RA patients (69 with rheumatoid factor (RF)^+^ RA and 36 with RF^−^ RA), the presence of AGE-damaged IgGs was examined in a follow-up investigation. ESR > 50 mm/hour and elevated IgG-AGE positive status were associated with an odds ratio of 3.19 (95% CI: 1.13–9.12; *p* = 0.0024). IgG-AGE and glucose levels did not correlate, proving that inflammation rather than hyperglycemia is what has the biggest impact on AGE generation in patients with early synovitis. IgG-AGE antibodies were highly substantially (*p* < 0.0001) correlated with RF presence, with hazard ratios of 9.54 (95% CI: 2.77–36.12). However, despite the fact that 10 patients (all of whom had RA) had high RF titers at the time of the initial visit but no anti-IgG-AGE response, this immune response to damaged IgG was not only a reflection of RF titer [55].

RA is linked to increased morbidity and mortality from cardiovascular disease (CVD), which is comparable with that in diabetics [47]. Traditional risk factors alone cannot explain the increase in morbidity and mortality [48]. Chronic inflammation is one of the non-traditional risk factors associated with RA, causing vascular endothelial dysfunction [49,50]. The endothelium is essential for a number of cardiovascular processes. Due to vasodilatation, inhibition of platelet aggregation, anticoagulant and profibrinolytic actions, and anti-inflammatory properties, it functions as an atheroprotective factor under normal conditions. Endothelial damage is a precursor of structural arterial wall damage and an early indicator of atherosclerosis, making it a useful benchmark for overall cardiovascular health. Whether endothelial damage in RA is related to AGE levels was investigated in a cross-sectional study involving 24 patients with established active RA (mean disease duration: 6.43+/−1.05 years) and high disease activity (DAS28 > 5.1) despite treatment with stable doses of conventional disease-modifying antirheumatic drugs (DMARDs). A substantial link between flow-mediated vasodilation and AGE levels was refuted by univariate correlation analysis (r = 0.33, *p* = 0.11) [50].

Increased AGE levels have been linked to the development of future microvascular and macrovascular events in both diabetics and non-diabetics [51,52,62]. The interaction between AGE and RAGE can contribute to the development of atherosclerosis [63,64,65]. In a cross-sectional study with 49 consecutive RA patients with longstanding disease (median disease duration: 12.3 years (range 9.3–15.1)) and 49 age- and sex-matched healthy controls, AGEs (as measured by skin AF) were increased in RA patients without signs of premature atherosclerosis. AGEs were related to endothelial activation and endothelial dysfunction, as demonstrated by an inverse relation with small artery elasticity and a positive relation to intima media thickness. This lends credence to the theory that AGEs may be an early indicator of cardiovascular disease in RA. There was no relationship between AGEs and disease activity or radiological damage, most likely because joint damage was quite low in this cohort, indicating a low disease activity throughout the course of the disease. In both groups, smoking, glucose levels, von Willebrand factor (vWF), small artery elasticity, and male gender were significantly related to the formation of AGEs in multivariate analysis [66].

### 2.2. The Relationship between (Soluble) Receptor for Advanced Glycation-End Products and Rheumatoid Arthritis

RAGE has been linked to the pathogenesis of RA due to its ability to increase inflammatory pathways (Table 1) [67,68]. Human synovial fibroblasts express RAGE, mainly in synovial intima [69]. A plethora of inflammatory RAGE ligands (such as S100A8/A9, S100A8 and S100A12) are present in active RA, both in the synovium [21,70,71,72] and in the circulation [21,73], modulating the expression of cell-bound RAGE. Anti-RAGE antibodies found locally in RA patients’ joints have been linked to a less destructive joint disease [74].

In contrast to previous studies [21,37,42,75], a cross-sectional study of 60 RA patients and 30 age- and sex-matched healthy controls, the serum sRAGE concentrations were significantly higher in RA patients than in healthy controls (*p* < 0.001). The serum sRAGE levels were affected by disease activity (*p* < 0.001). Disease activity, ESR, and CRP all correlated significantly with the serum sRAGE concentrations (r = 0.67, *p* = 0.001; r = 0.411, *p* = 0.001; r = 0.273, *p* = 0.035) [76]. Although, in another cross-sectional study, CRP, ESR, and disease activity scores did not substantially correlate with serum sRAGE levels. This casts doubt on the utility of sRAGE as a biomarker for disease activity [77].

Furthermore, no significant correlations were found between serum sRAGE and demographic or biochemical measurements such as serum creatinine, blood urea nitrogen (BUN), RF, or anti-cyclic citrullinated peptides (anti-CCP). When disease activity is controlled, the increase in serum sRAGE is most likely due to a reduction in proinflammatory and/or proatherogenic RAGE. Increased sRAGE production and release in RA patients with high disease activity may be a compensatory anti-inflammatory mechanism in response to tissue damage, as it has the ability to bind ligands and prevent them from activating detrimental signaling pathways. As an immune surveillance mechanism, sRAGE may block the ligand–RAGE interaction on the cell surface, reducing the influx of inflammatory cells into the joint cavity. A decreased level of sRAGE in RA patients might increase the tendency toward inflammation [76]. In order to consider serum sRAGE variations as an active marker for RA, a longitudinal analysis will be more useful.

Data from 342 consecutive early RA patients participating in the ‘Parelsnoer’ cohort showed that suppressing inflammation (demonstrated by a decrease in ESR or CRP), independently of achieving remission, increases sRAGE levels significantly. There was a 2.2-point increase in sRAGE for every point decrease in ESR and a 1.7-point decrease for every point decrease in CRP, respectively (*p* < 0.001) [78].

The increased CVD rate in RA patients appears to be related to sRAGE levels as well [16,65,75]. In female RA patients, low serum sRAGE levels were significantly associated with previous history and new imminent cardiometabolic events. This low sRAGE was attributed primarily to negative metabolic parameters (including high fasting plasma glucose and body fat content) rather than signs of inflammation, and the association was prominent in patients younger than 50 years. Low sRAGE indicates a metabolic imbalance prior to clinical cardiometabolic events. Besides inflammation, the presence of hyperglycemia may be another molecular explanation for decreased sRAGE. Hyperglycemia causes an increased production of AGEs. sRAGE binds to AGEs without activating cellular pathways and acts as a decoy for AGEs, increasing consumption while decreasing detectable circulating levels. Binding AGEs, on the other hand, blocks AGE-RAGE signaling, reduces the positive feedback loop for RAGE upregulation, and thus potentially limits RAGE enzymatic cleavage. Based on these findings, sRAGE could be used to monitor cardiometabolic health in RA patients [16]. Further, in a cohort of early RA patients, a prospective study investigated the relationship between serum levels of sRAGE and the progression of carotid atherosclerosis and arterial stiffness measures. Aortic augmentation index (AIx) and pulse wave velocity (PWV) both significantly decreased after 12 months of efficient inflammation control (*p* = 0.001 and *p* < 0.05, respectively), while sRAGE levels increased significantly (*p* < 0.001). In this study, a drop in AIx was significantly correlated with an increase in sRAGE levels (r = 0.259, *p* = 0.023). Therefore, sRAGE may be crucial in the way that the interaction between ligand and RAGE causes inflammation and vascular stiffness [75]. Reduced levels of sRAGE may lead to arterial stiffness through a number of different processes. First, it has been demonstrated that RAGE expression is associated with inflammatory markers [79], which may reduce the endogenous vasodilator nitric oxide, a crucial functional regulator of major artery stiffness in vivo [80]. Second, lower sRAGE levels may obstruct the vascular wall’s extracellular matrix’s capacity to renew its elastin and collagen [80,81]. In a Japanese study, the role of RAGE in the pathophysiology of RA was examined. Utilizing immunohistochemical labeling, RAGE expression in synovial tissues was evaluated. IL-17 or IL-1 treatment boosted RAGE expression and production in RA-fibroblast-like synoviocytes (*p* < 0.05 vs. untreated cells). In comparison to a single stimulus with IL-17 or IL-1 alone, the combined stimuli of both IL-17 and IL-1 significantly boosted RAGE production (*p* < 0.05 vs. 10 ng/mL IL-17). The increased RAGE generation brought on by IL-17 was totally reduced by the addition of Act-1 shRNA to the RA-FLS culture supernatant. This investigation revealed that IL-17 plays a significant role in the up-regulation of RAGE in RA-fibroblast-like synoviocytes. Since Act-1 is thought to be involved in IL-17-induced RAGE up-regulation, targeting Act-1 may be a viable tactic for regulating RAGE expression [82].

sRAGE also has therapeutic properties in inflammatory diseases because it reduces the immune inflammatory response caused by HMGB-1. Moreover, mesenchymal stem cells (MSCs) are appealing agents for RA cellular therapy [83]. Although MSCs are immunosuppressive agents in inflammatory disorders, they also play a role in the inflammatory process and the induction of immune responses [84]. In an experimental murine model of inflammatory arthritis, sRAGE-MSCs were generated by DNA transfection of human adipose tissue-derived MSCs (Ad-hMSCs). sRAGE-MSCs produced significantly less proinflammatory molecules (vascular endothelial growth factor (VEGF), interleukin-1 (IL-1), IL-6, and HMGB-1) in comparison with MSCs under inflammatory conditions. sRAGE-MSCs attenuated rheumatoid inflammation through reciprocal regulation of Th17 and Treg cells. These findings suggest that sRAGE overexpression in Ad-hMSCs can inhibit inflammation and promote the immunoregulatory function while preserving their unique properties [83].

**Table 1:** Overview of studies investigating advanced glycation end products (AGEs) and the (soluble) receptor for AGEs ((s)RAGE) in rheumatoid arthritis.

Parameter	Study Design	Study Population	Major Findings	Ref.
Serum pentosidine	Cross-sectional study	60 RA patients 37 SLE patients 61 diabetics 57 HC	RA vs. HC: *p* < 0.0001	[38]
	Cross-sectional study	133 non-diabetic RA patients 56 age-matched HC	RA vs. HC: *p* < 0.05 Correlation to ESR: r = 0.226, *p* = 0.011 Correlation to CRP: r = 0.208, *p* = 0.022	[39]
	Cross-sectional study	80 RA patients 30 HC	RA vs. HC: *p* < 0.0001 RA with HDA vs. MDA: *p* < 0.001	[40]
	Cross-sectional study	39 RA patients 38 HC	RA vs. HC: *p* = 0.03 Correlation with synovial pentosidine: r = 0.97, *p* < 0.001 Correlation with DAS28: r = 0.02, *p* = NS Correlation with ESR: r = 0.44, *p* < 0.03 Correlation with CRP: r = 0.04, *p* = NS	[53]
	Follow-up cohort study	22 RA patients	Pretreatment vs. after 6 months etanercept: *p* < 0.05 Correlation with number of swollen joints: r = 0.449, *p* < 0.05 Correlation with number of tender joints: r = 0.489, *p* < 0.05	[54]
Urinary pentosidine	Follow-up cohort study	22 RA patients	Pretreatment vs. after 6 months etanercept: *p* < 0.05 Correlation with DAS28: r = 0.395, *p* < 0.05 Correlation with number of swollen joints: r = 0.396, *p* < 0.05 Correlation with number of tender joints: r = 0.418, *p* < 0.05	[54]
Synovial pentosidine	Cross-sectional study	39 RA patients 38 HC	Correlation with serum pentosidine: r = 0.97, *p* < 0.001	[53]
Serum CML	Cross-sectional study	80 RA patients 30 HC	RA vs. HC: *p* < 0.0001 RA with HDA vs. MDA: *p* < 0.001 RA risk: OR = 1.21, 95% CI: 1.05–1.39, *p* = 0.006 MLR with DAS28: β = 0.43, *p* = 0.001 Severity prediction: 88% sensitivity and 82% specificity	[40]
	Cross-sectional study	559 women of ≥65 years old	All case mortality in Q4 (>0.68 μg/mL): HR = 1.47, 95% CI: 0.97–2.22 Q4 CVD death: 18.9% Decreased survival Q4 vs. Q1–Q3: *p* = 0.0009 Death due to CVD Q4 vs. Q1–Q3: HR: 1.94, 95% CI: 1.08–3.48, *p* = 0.026	[62]
Synovial CML	Cross-sectional study	10 RA patients 8 controls (4 healthy, 4 OA)	CML present in the synovial lining, sublining, and endothelium Macrophages (CD68^+^) and T-cells (CD45RO^+^): +immunostaining B-cells: –immunostaining	[41]
Serum MGO	Cross-sectional study	80 RA patients 30 HC	RA vs. HC: *p* < 0.0001 RA with HDA vs. MDA: *p* = 0.019 RA with HDA vs. HC: *p* = 0.004 RA with MDA vs. HC: *p* = 0.002 RA risk: OR = 1.17, 95% CI: 1.02–1.31, *p* = 0.01	[42]
Skin AGE	Cross-sectional study	93 RA patients 24 OA patients 29 DRSA patients 43 HC	RA vs. HC: significant * Correlation with age: r = 0.25 and *p* = 0.015	[46]
	Cross-sectional study	49 consecutive RA patients with longstanding disease 49 age- and sex-matched HC	RA vs. HC: *p* = 0.03	[66]
Serum IgG-AGE	Prospective cohort study	69 RF^+^ RA patients 39 RF^−^ RA patients 43 SpA patients 90 UA	RA vs. SpA: *p* < 0.05 RA vs. UA: *p* < 0.05 Correlation with ESR > 50 mm/hour: OR = 3.19, 95% CI: 1.13–9.12; *p* = 0.0024 Correlation with RF presence: OR = 9.54, 95% CI: 2.77–36.12, *p* = 0.0001	[55]
Serum sRAGE	Prospective follow-up cohort study	171 female RA patients CMRG (*n* = 13; CVD high risk: sRAGE ≤ Q3; CVDlow risk: > Q4)	sRAGE CVD high risk group vs. CMRG: *p* < 0.0001 CVD high risk group correlation with DAS28: *p* < 0.05 CVD high risk group correlation with number of tender and swollen joints: *p* < 0.05	[16]
	Cross-sectional study	559 women of ≥65 years old	All-case mortality: HR = 1.19, 95% CI: 0.98–1.44, *p* = 0.07 CVD mortality: HR = 1.27, 95% CI: 0.98–1.65, *p* = 0.07	[62]
	Cross-sectional study	138 patients with established RA 44 HC	RA vs. HC: *p* = NS Linear regression with S100A9: β = −0.77, 95% CI: −0.78 to 0.009, *p* = 0.06 Linear regression with S100A8: β = −0.39, 95% CI: −1.37 to −016, *p* = 0.01 Linear regression with S100A12: β = −0.28, 95% CI: −0.61 to 0.05, *p* = 0.09	[21]
	Prospective cohort study	94 early RA patients	Increase in sRAGE levels after 12 months: *p* < 0.001 Correlation with change in Aix: r = −0.259, *p* = 0.023	[75]
	Cross-sectional study	60 RA patients 30 age- and sex-matched HC	RA vs. HC: *p* < 0.001 Correlation with DAS28: r = 0.67, *p* < 0.001 Correlation with ESR: r = 0.411, *p* = 0.001 Correlation with CRP: r = 0.273, *p* = 0.035 Correlation with serum creatinine, BUN, RF, and anti-CCP: *p* = NS	[76]
	Cross-sectional study	20 patients with Behcet disease 20 RA patients 20 SLE patients 22 HC	RA vs. HC: *p* < 0.001 Correlation with CRP: r = −0.163, *p* = NS Correlation with ESR: r = −0.387, *p* = 0.092 Correlation with disease activity scores: r = −0.016, *p* = NS	[77]
	Follow-up cohort study	342 early RA patients	Increase in sRAGE levels after 6 months: *p* < 0.001	[78]
Blood sRAGE	Cross-sectional study	62 RA patients 33 NID 45 HC	RA vs. HC: *p* < 0.001 RA vs. NID: *p* = 0.0025 Correlation with synovial sRAGE: r = 0.48, *p* = 0.002	[37]
Synovial sRAGE	Cross-sectional study	14 RA patients 7 OA patients	RA vs. OA: *p* < 0.05	[67]
	Cross-sectional study	62 RA patients 33 NID 45 HC	RA vs. NID: *p* = NS Correlation with blood sRAGE: r = 0.48, *p* = 0.002	[37]
	Cross-sectional study	Human FLSs isolated from synovial tissues from RA and OA patients *	Strong expression in RA synovial tissues Increase in sRAGE with IL-17 treatment at 10 ng/mL vs. untreated cells: *p* < 0.05 Increase in sRAGE with IL-1β treatment vs. untreated cells: *p* < 0.05 Change of sRAGE with anti-TNF-α treatment vs. untreated cells: *p* = NS	[82]

Abbreviations: Aix: aortic augmentation index; CI: confidence interval; CML: carboxymethyllysine; C-reactive protein; DAS: disease activity score; ESR: erythrocyte sedimentation rate; DRSA: destructive spondyloarthropathy; HC: healthy control subjects; HDA: high disease activity: IL, interleukin; MDA: moderate disease activity; MGO: methylglyoxal; MLR: multiple logistic regression; NID: non-inflammatory joint disease patients; NS: not significant; OA: osteoarthritis; OR: odds ratio; RA: rheumatoid arthritis; SpA: spondyloarthropathy; TNF-α: tumor necrosis factor-alpha; UA: undifferentiated arthritis; CMRG: cardiometabolic reference group (female RA patients with diagnosed cardiometabolic diseases/events). * not further specified.

## 3. Systemic Lupus Erythematosus

Systemic lupus erythematosus (SLE) is a pleiomorphic, autoimmune disorder characterized by chronic inflammation and immune complex formation in the connective tissue of multiple organs. This results in a heterogeneous clinical presentation with common involvement of the skin, kidneys, joints, and nervous system. Non-white women between the ages of 20 and 40 are the group with the highest prevalence of this illness [85,86,87,88]. The exact underlying pathophysiology remains unknown. SLE is considered as a multifactorial disease determined by several genetic and environmental factors [86].

### 3.1. The Importance of Advanced Glycation-End Products in Systemic Lupus Erythematosus

Chronic inflammation in SLE appears to be associated with an intensified glycation process and the formation of AGEs. Many researchers looked into the significance of AGEs in SLE patients (Table 2) [38,89,90,91,92,93]. A cross-sectional study in 55 inactive SLE patients and 55 controls, who were of similar age and sex, examined whether elevated levels of AGEs were present in patients with SLE by monitoring UV-A light excitation–emission matrices (AF-EEMS). When compared to controls, AF-EEMS were significantly higher in SLE patients (1.50 0.5 a.u. vs. 1.28 0.4 a.u., *p* = 0.006) [93]. In another study of 31 female SLE patients and 26 healthy female control subjects, a higher serum concentration of AGEs and a lower serum concentration of sRAGE was measured in the patient group. Serum CML, N^ε^-carboxy-ethyl-lysine (CEL), and pentosidine concentrations did not differ between groups in a statistically significant way. So, SLE patients may be at risk of accelerated glycation and activation of the proinflammatory RAGE, which may worsen the disease’s course [22].

The increased AGE accumulation is related to accelerated atherosclerosis in SLE [92,93,94]. The association between AGE levels and the small arterial elasticity was studied in a cross-sectional study with 30 SLE patients and 30 age- and sex-matched healthy controls (SAE). This led to a moderate correlation that was statistically significant (r = −0.370, *p* < 0.05) [92]. This relationship may be explained by three general mechanisms. First, the flexibility of blood vessels is reduced as a result of AGEs’ cross-linking with extracellular matrix proteins. Second, the creation of intracellular AGE may change cellular function. Third, through interacting with and activating RAGE and other receptors, AGEs may modify how cells function. Regrettably, it is unknown which specific compounds contribute to the overall increase in AGEs’ concentration in SLE patients [89]. Although suggested by some authors [22], CML and CEL concentrations did not reflect total AGE concentrations in blood [89]. Despite being AGEs, these compounds cannot be used as markers of the glycation process in SLE patients. Other AGEs, such as glycolic acid lysine amide (GALA), glyoxal lysine amide (GOLA), glyoxal lysine dimer (GOLD), and MOLD, should be studied [95].

### 3.2. The Relationship between (Soluble) Receptor for Advanced Glycation-End Products and Systemic Lupus Erythematosus

The serum sRAGE concentrations are lower in SLE patients in comparison to healthy controls (Table 2) [22,89,90,91,96,97,98,99,100]. There are two possibilities for the concurrent decrease in sRAGE concentration and increase in AGE concentration. A primary phenomenon could be a sRAGE deficiency, allowing more AGEs to remain unbound in body fluids. It is also possible that the deficit is a secondary phenomenon, as the amount of sRAGE could be depleted by excessively generated AGEs or other ligands of this receptor. Whatever the cause, sRAGE deficiency may contribute to more frequent interactions between AGEs and transmembrane RAGE. In the murine model, it was discovered that sRAGE could prevent the activation of proinflammatory pathways [101]. In the study, a cumulative analysis of all participants in both groups revealed a weak, positive correlation between AGE concentration in serum and smoking duration (but no correlation between AGE concentration and participant age). As a result, smoking may contribute to an increase in the accumulation of AGEs [89].

Similar to diabetic and hypertensive patients, a Chinese cross-sectional study demonstrated that arterial stiffness in female SLE patients is thought to be inversely correlated with sRAGE. An automatic pulse wave analyzer was used to assess the brachial–ankle pulse wave velocity (baPWV). According to the baPWV values, the patients were split into two groups; those who had values >1400 cm/s were assigned to the group with excessive arterial stiffness (*n* = 35). In the group with high arterial stiffness, sRAGE levels were significantly lower (*p* < 0.05). According to a linear regression analysis, the levels of sRAGE were substantially correlated with baPWV (standardized = 1.18, *p* < 0.01). In these patients, arterial stiffness was independently predicted by sRAGE, SLE duration, systolic blood pressure, and low-density lipoprotein cholesterol, according to multivariate logistic regression analysis [102].

Another Chinese, cross-sectional study looked at the amount of sRAGE in various clinical forms of lupus nephritis (LN) in order to determine the usefulness of these biomarkers for predicting the prognosis of the disease. First, the proliferative group of LN showed significantly higher serum RAGE levels than the non-proliferative group (*p* = 0.0053). This contributes to our understanding of the many aspects of LN and may aid in determining the kind of disease before biopsy and throughout follow-up. Additionally, the group that had a poor response to immunosuppressant therapy, the serum RAGE level was 1141.64+/−828.53 pg/mL, while in the group that had a favorable response, it was 1700.42+/−1345.38 pg/mL. This variation in serum RAGE levels among the various groups was significant (*p* = 0.0055). The effectiveness of indicators for predicting the prognosis of LN was evaluated using logistic regression. Serum RAGE has a significance level of *p* = 0.043, suggesting lower blood RAGE levels may be an independent risk factor for a suboptimal response to immunosuppressive medication [103]. RAGE polymorphisms may be linked to SLE and LN susceptibility [27,104,105].

Advanced glycation end-product-specific receptor (*AGER*) is a gene that encodes the cell surface receptor RAGE. This gene can be found at 6p21.3 on the short arm of chromosome 6 [1]. This locus is the site of major histocompatibility complex III and is implicated in inflammatory and immunological responses [105]. In a cross-sectional study, DNA samples of 97 SLE patients, 114 LN patients, and 429 healthy controls were genotyped for four RAGE polymorphisms: −429 T/C, −374 T/A, 2184 A/G, and Gly82Ser. When compared to healthy controls, the C allele of −429 T/C, the T allele of −374 T/A, and the G allele of 2184 A/G were significantly more prevalent in SLE and LN. During the first two years of treatment, the C allele of RAGE −429 T/C, the A allele of RAGE −374 T/A, and the G allele of RAGE 2184 A/G polymorphism were significantly associated with more proteinuria and worse renal function in LN. There was no evidence of a genotype–sRAGE relationship [14]. Both the 429 T/C and 374 T/A polymorphisms had a significant impact on *RAGE* gene expression. It has been proposed that the A allele of 374 T/A causes less RAGE expression, reducing the inflammatory response [27,104]. The presence of the G allele of 2184 A/G may result in increased oxidative stress, possibly leading to inflammation [106]. The RAGE polymorphisms discovered are linked to SLE susceptibility in general, but not to renal involvement in particular [14].

**Table 2:** Overview of studies investigating advanced glycation end products (AGEs) and the (soluble) receptor for AGEs [(s)RAGE] in systemic lupus erythematosus.

Parameter	Study Design	Study Population	Major Findings	Ref.
Serum pentosidine	Cross-sectional study	60 RA patients 37 SLE patients 61 diabetics 57 HC	SLE vs. HC: *p* = NS	[38]
	Cross-sectional study	31 adult, female SLE patients 26 age-matched female HC	SLE vs. HC: *p* = NS Correlation with CEL: r = 0.53, *p* < 0.01 Correlation with SLEDAI-2K: *p* = NS	[89]
	Cross-sectional study	38 SLE patients LN 44 SLE patients non-LN 40 HC	SLE-LN vs. SLE-non-LN: *p* = 0.05 SLE-LN vs. HC: *p* = 0.006 SLE-non-LN vs. HC: *p* = 0.009	[90]
Serum CML	Cross-sectional study	31 adult, female SLE patients 26 age-matched female HC	SLE vs. HC: *p* = NS Correlation with SLEDAI-2K: *p* = NS	[89]
Serum CEL	Cross-sectional study	31 adult, female SLE patients 26 age-matched female HC	SLE vs. HC: *p* = NS Correlation with pentosidine: r = 0.53, *p* < 0.01 Correlation with SLEDAI-2K: NS	[89]
Serum AGE	Cross-sectional study	31 adult, female SLE patients 26 age-matched female HC	SLE vs. HC: *p* < 0.01 Correlation with SLEDAI-2K: *p* = NS	[89]
	Cross-sectional study	38 SLE patients LN 44 SLE patients without LN 40 HC	SLE-LN vs. SLE-non-LN: *p* = 0.08 SLE-LN vs. HC: *p* = 0.008 SLE-non-LN vs. HC: *p* = 0.005	[90]
Plasma AGE	Cross-sectional study	52 AOSD patients 36 SLE patients 16 HC	SLE vs. HC: *p* < 0.001 AOSD vs. HC: *p* < 0.001	[91]
Skin AGE	Cross-sectional study	55 SLE patients with inactive disease 55 age- and sex-matched HC	SLE vs. HC: *p* = 0.006 Patients with manifest CVD vs. without manifest CVD: *p* = NS	[93]
	Cross-sectional study	30 SLE patients 30 age- and sex-matched HC	SLE vs. HC: *p* = 0.02 Correlation with SAE: r = −0.370, *p* < 0.05	[92]
Serum sRAGE	Cross-sectional study	20 patients with Behcet disease 20 RA patients 20 SLE patients 22 HC	SLE vs. HC: *p* < 0.001 Correlation with CRP: r = 0.194, *p* = NS Correlation with ESR: r = 0.426, *p* = NS Correlation with disease activity scores: r = 0.057, *p* = NS	[77]
	Cross-sectional study	31 adult, female SLE patients 26 age-matched female HC	SLE vs. HC: *p* < 0.05 Correlation with SLEDAI-2K: NS	[89]
	Cross-sectional study	38 SLE patients LN 44 SLE patients without LN 40 HC	SLE-LN vs. SLE-non-LN: *p* = 0.06 SLE-LN vs. HC: *p* = 0.0008 SLE-non-LN vs. HC: *p* = 0.001	[90]
	Cross-sectional study	97 JIA children 19 SLE children 27 HC	SLE vs. HC: *p* < 0.05 JIA vs. HC: *p* = 0.006	[97]
	Cross-sectional study	60 APS patients 30 HC	APS patients vs. HC: *p* < 0.0001	[100]
	Cross-sectional study	82 proliferative LN patients 53 non-proliferative LN patients 43 mixed LN patients	Proliferative LN vs. non-proliferative LN vs. mixed LN: *p* = 0.0053 Good vs. bad response on immunosuppressive therapy: *p* = 0.0055 Correlation with SLEDAI-2K: r = 0.12, 95%CI: −0.02454 to 0.2653, *p* = 0.11	[103]
Plasma sRAGE	Cross-sectional study	52 AOSD patients 36 SLE patients 16 HC	SLE vs. HC: *p* < 0.001 AOSD vs. HC: *p* < 0.001	[91]
	Cross-sectional study	105 SLE patients 43 HC	Active SLE vs. HC: *p* = 0.003 Non-active SLE vs. HC: *p* = 0.012 Active SLE vs. non-active SLE patients: NS Treated SLE vs. HC: *p* = 0.004 Untreated SLE vs. HC: *p* = 0.005 Treated vs. untreated SLE: *p* = 0.782	[96]
	Cross-sectional study	11 pAPS patients 17 APA + SLE patients without APS manifestations (APA + SLE) 12 SLE patients with secondary APS (APS + SLE) 10 HC	APA + SLE vs. HC: *p* = NS APS ^+^ SLE vs. HC: *p* = NS APS + SLE vs. APA + SLE: *p* = NS Correlation with SLEDAI-2K: *p* = NS	[98]
	Cross-sectional study	27 female SLE-LN+ patients 24 female HC	SLE-LN+ with flare vs. HC: *p* = NS SLE-LN+ without flare vs. HC: *p* < 0.05 SLE-LN+ with flare vs. SLE-LN+ without flare: *p* < 0.001 Correlation with SLEDAI-2K: r = −0.483, *p* = 0.011	[99]
	Cross-sectional study	94 female SLE patients (2 groups: HAS: BaPWV > 1400 cm/s (*n* = 35); LAS: BaPWV ≤ 1400 cm/s (*n* = 59)	HAS vs. LAS: *p* < 0.05 Correlation with BaPWV: β = 1.18, *p* < 0.01	[102]

Abbreviations: AOSD: adult-onset Still’s disease; APA: antiphospholipid antibody; APS: antiphospholipid syndrome; BaPWV: brachial–ankle pulse wave velocity; CEL: carboxyethyllysine; CML: carboxymethyllysine; HAS: high arterial stiffness; HC: healthy controls; JIA: juvenile idiopathic arthritis; LAS: low arterial stiffness k; LN: lupus nephritis; NS: non-significant; pAPS: primary antiphospholipid syndrome; RA: rheumatoid arthritis; SAE: small arterial elasticity; SLE: systemic lupus erythematosus; SLEDAI-2K: Systemic Lupus Erythematosus Disease Activity Index 2000.

## 4. Sjögren’s Syndrome

Sjögren’s syndrome (SS) is a chronic autoimmune disease characterized by sicca symptoms caused by lymphatic infiltration and secondary exocrine gland damage. Other severe complications, ranging from interstitial lung disease to vasculitis and central nervous system involvement, occur in addition to this characteristic manifestation. Some SS patients exhibit symptoms very similar to SLE, and there is overlap between these two entities in terms [107]. The RAGE system may be involved in the primary SS disease pathway, and sRAGE may be a potential biomarker to aid in the diagnosis of primary SS [108]. RAGE is overexpressed on the epithelial duct cells of SS patients’ minor salivary glands, which could also indicate increased signaling through the receptors. Accumulation of AGEs in the salivary glands of SS patients via binding and activation of RAGE may initiate an inflammatory cell infiltrate, which can mediate [109].

### The Relationship between (Soluble) Receptor for Advanced Glycation-End Products and Sjögren’s Syndrome

In a cross-sectional study with 39 SS patients and 21 healthy controls, significantly higher serum sRAGE concentrations were measured in the patient group (*p* = 0.003), which correlated with the European League Against Rheumatism (EULAR) SS disease activity index (ESSDAI) (r = 0.545, *p* = 0.002) [110]. A preliminary data study demonstrated that RAGE is present in both normal and SS labial salivary glands. SS samples expressed approximately 100% more RAGE than controls in immunoblotting (*p* < 0.03) [109]. In a small cross-sectional study, primary SS patients had significantly lower serum sRAGE concentrations than secondary SS patients and patients with only a positive ANA titer. The level of sRAGE was a significant predictor of diagnostic category. The odds ratio for primary SS diagnosis was 6.03, indicating that the probability of a primary Sjögren’s diagnosis increased by a factor of six for each 300 pg/mL decrease in sRAGE levels [108]. The relative decrease in sRAGE in primary SS serum could explain the immune system’s reduced ability to negate this effect by binding sRAGE to RAGE ligands and preventing inflammatory signaling via the cellular RAGE. Furthermore, cell-bound RAGE acts as a counter-receptor for leukocyte integrins, resulting in direct involvement in leukocyte recruitment, particularly in inflammatory conditions when receptor expression increases [111].

## 5. Adult-Onset Still’s Disease

Adult-onset Still’s disease (AOSD) is a rare, autoinflammatory disorder characterized by fever, rash, arthritis, multiorgan involvement, and increased acute phase reactants [112]. Although the exact etiology of AOSD is unknown, cytokine-mediated inflammation may play a role in its development [113].

### 5.1. The Importance of Advanced Glycation-End Products in Adult-Onset Still’s Disease

Active AOSD patients had significantly higher median AGE levels than healthy subjects (*p* < 0.001). Overproduction of AGEs may be involved in the pathogenesis of AOSD and may act as disease activity indicators. In AOSD patients, plasma AGE concentrations were positively correlated with activity scores (r = 0.836, *p* < 0.001), ferritin levels (r = 0.372, *p* < 0.05), and CRP levels (r = 0.396, *p* < 0.005). In comparison to AOSD patients with monocyclic patterns, those with polycyclic or chronic articular patterns had significantly higher plasma AGE concentrations, which may be due to cumulative inflammation. AGE levels decreased with treatment [91]. The presence of anemia in AOSD patients was associated with AGE levels, which is consistent with previous findings that plasma AGEs, an indicator of oxidative stress, were a predictor of anemia in older community-dwelling adults [114]. As AGEs are a heterogeneous group of compounds, more research is needed to identify the major compound linked to AOSD [91].

### 5.2. The Relationship between Soluble Receptor for Advanced Glycation-End Products and Adult-Onset Still’s Disease

Active AOSD patients had significantly lower median sRAGE concentrations than healthy controls (*p* < 0.001) [91,115]. Inadequate sRAGE levels may exacerbate inflammatory responses by increasing the engagement of accumulated AGEs on cell-bound RAGE. AOSD activity scores were negatively correlated with plasma sRAGE levels (r = −0.320, *p* < 0.05). sRAGE levels increased in tandem with the decrease in disease activity [91]. However, this relationship was not always demonstrated [115]. Plasma sRAGE may serve as a biomarker and negative regulator of inflammation [3], and recombinant sRAGE administration has been shown to be therapeutic in inflammatory murine models [116,117]. The low plasma sRAGE levels in active AOSD patients may be explained by its binding with inflammatory ligands, promoting sRAGE consumption [118]. In this study, elevated AGE levels may increase the binding and consumption of sRAGE, which is consistent with this hypothesis. The other possibility is that the decreased production of endogenous secretory sRAGE is insufficient as a decoy receptor for binding inflammatory ligands, resulting in weakened protection against the inflammatory response [37]. A logistic regression analysis demonstrated a negative association with the presence of leukocytosis. Acting as a counter-receptor for the leukocyte integrin Mac-1, cell-bound RAGE is involved in leukocyte recruitment. sRAGE could be a potential inhibitor of leukocyte recruitment [111]. Plasma sRAGE concentrations were lower in polycyclic AOSD patients than in monocyclic AOSD patients. A dysregulated inflammation in polycyclic pattern patients may persist due to insufficient inhibition by sRAGE. Long-term follow-up revealed that sRAGE levels increased with therapy, paralleling the decrease in disease activity [91]. The change in sRAGE concentrations after a 6-month therapy was comparable to a recent discovery that sRAGE levels increased after long-term (more than one month) treatment [96].

## 6. Juvenile Idiopathic Arthritis

Juvenile idiopathic arthritis (JIA) is a chronic disease is a heterogeneous group of arthritis diseases with the onset before 16 years of age and persistent for more than 6 weeks. It is characterized by inflammation and articular damage [119]. Systemic JIA (sJIA) differs from oligoarticular or polyarticular subtypes in that it is characterized by systemic and inflammatory features [120]. Although the etiology and pathogenesis of JIA are unknown, innate immunity is thought to play a role in disease persistence. Exogenous (microbial products) [121] or endogenous (damage-associated molecular patterns) triggers can be used (e.g., S100A12). They stimulate immune responses via pattern recognition receptors, of which the mRAGE is one [122].

### The Relationship between (Soluble) Receptor for Advanced Glycation-End Products and Juvenile Idiopathic Arthritis 

Children with JIA were characterized by significantly lower sRAGE levels compared to the healthy controls (*p* = 0.006). An inverse correlation between inflammatory markers (serum HMGB1, ESR, CRP, α2 globulin) and serum sRAGE concentrations was found. As also significantly higher HMGB1 concentrations were detected in all subtypes of JIA patients versus healthy controls (*p* < 0.001), inflammation triggered by alarmins plays a role in pathogenesis of JIA [97].

Patients with JIA-enthesitis-related arthritis (ERA) had significantly lower serum sRAGE concentrations than healthy controls (*p* < 0.0001). In paired samples, synovial fluid levels were lower than plasma levels (*p* < 0.0001). This reflects the important regulatory role of sRAGE. Lower sRAGE levels in synovial fluid could lead to continued activation of RAGE. Serum sRAGE concentrations were negatively correlated with S100A12 levels (r = −0.474; *p* < 0.01), ESR (r = −0.306; *p* < 0.01), and swollen joint count (r = −0.237; *p* < 0.05), but not with tender joint count. In patients with stable disease, sRAGE levels remained stable over time [122].

The *AGER* rs1035798 AA genotype was found to be a risk factor in a study of 201 Belarusian children with JIA. Allele A was also linked to JIA (*p* = 0.0058) and articular syndrome of non-autoimmune origin (*p* = 0.0264). The AA genotype was found in the highest frequency in JIA patients with polyarthritis or severe oligoarthritis. However, the functional significance of the rs1035798 polymorphism remains unknown [123].

## 7. Conclusions

Until now, the greatest challenge is to determine in detail the potential role of the AGE-RAGE axis signaling pathways and sRAGE in the multifactorial origin of rheumatic diseases. Although the importance of AGEs in RA and SLE has already been demonstrated in the current review, there is a push to investigate the role of AGEs in the pathogenesis of SS, AOSD, and JIA. Due to the heterogeneous nature of AGEs and the limitations of available analysis techniques, there are currently no standardized procedures to measure AGEs [124]. As a result, the use of AGEs in diagnosis of rheumatic diseases is still restricted. Moreover, it is often unknown which compounds contribute to the increase in total AGE concentrations in the blood or which could be considered potential markers of the glycation process in the several discussed pathologies. Regardless of the role of AGEs in the initiation or progression of rheumatic disorders, lowering its level is unquestionably beneficial to health.

Moreover, it is unclear whether elevated or decreased serum sRAGE concentrations are linked to risk factors or illness conditions, which is the most perplexing element of sRAGEs. There is not always a pretest likelihood for the direction of connections, which has been recorded together with many post hoc comments. This suggests that there is not a single, underlying theory explaining how sRAGEs alter in response to risk factors or disease conditions, such as increased or decreased production (up- or downregulation), disturbed elimination due to low eGFR, competition between different RAGEs, or interruption of the ligand–RAGE pathway.

As several studies presented in this review have a cross-sectional design, there is a call for longitudinal studies to evaluate the usefulness of sRAGE as a biomarker taking into account the different sRAGE isoforms. When evaluating the levels of circulating sRAGE, the influence of several drugs should be taken into account. Multiple studies have shown that treatment for hypertension (e.g., ACE inhibitors) and hyperlipidemia [125,126], as well as DMARD treatment with methotrexate, can modulate sRAGE levels. Moreover, the relationship between serum sRAGE concentrations and clinical parameters should be further explored.

Finally, novel insights might lead to the development of inhibitors that act on different steps of the AGE-RAGE axis. In this regard, sRAGE has emerged as a promising molecule in multiple experimental studies to competitively inhibit RAGE activation, modulating the systemic inflammation caused by the AGE-RAGE axis.

## Data Availability

Not applicable.
